# Pharmacokinetic analysis of morphine-3-glucuronide after acute morphine intravenous bolus administration to rats with traumatic brain injury

**DOI:** 10.1016/j.jpet.2025.103645

**Published:** 2025-06-25

**Authors:** Jonathan Birabaharan, Jeremy Henchir, Sarah Svirsky, Thomas D. Nolin, Philip E. Empey, Shaun W. Carlson

**Affiliations:** 1 Department of Pharmaceutical Sciences, School of Pharmacy, Center for Clinical Pharmaceutical Sciences, University of Pittsburgh, Pittsburgh, Pennsylvania; 2 Department of Neurological Surgery, University of Pittsburgh Medical Center, Pittsburgh, Pennsylvania; 3 Department of Pharmacy and Therapeutics, School of Pharmacy, University of Pittsburgh, Pittsburgh, Pennsylvania; 4 Center for Clinical Pharmaceutical Sciences, School of Pharmacy, University of Pittsburgh, Pittsburgh, Pennsylvania

**Keywords:** Glial fibrillary acidic protein, M3G, Morphine, Neuroinflammation, Pharmacokinetics, Traumatic brain injury

## Abstract

This study investigates the effects of traumatic brain injury (TBI) on the pharmacokinetics of morphine and its metabolite, morphine-3-glucuronide (M3G), and their influence on neuroinflammation and systemic inflammation. We hypothesized that disruptions in the blood–brain barrier (BBB) due to TBI would enhance M3G exposure to the brain, which could potentially trigger inflammatory responses. We implemented a rat model of controlled cortical impact (CCI) injury to assess systemic pharmacokinetics of morphine and M3G over 24 hours postintravenous bolus administration. To gain an understanding of relative levels in the brain, we measured the drug and metabolite concentrations in both brain tissue and plasma at the systemic maximum concentration, which occurred at 1 hour post-CCI. While this study was designed to conduct a thorough acute pharmacokinetic analysis, the design also afforded an early examination of potential pharmacodynamic effects. Markers of neuroinflammation and systemic inflammation were measured in plasma and cerebrospinal fluid at 24 hours post-CCI. Results showed a 2-fold increase in systemic M3G exposure and doubled concentrations of both morphine and M3G in the brain. Notably, only M3G demonstrated a significant increase in the brain/plasma ratio at 1 hour. Despite these pharmacokinetic changes following a single bolus, there were limited morphine-induced or M3G-induced increases in markers of neuroinflammation or systemic inflammation at 24 hour post-CCI. This study highlights that TBI significantly alters the pharmacokinetics of morphine and M3G, increasing their brain penetration without worsening acute inflammation. Future research will need to explore the implications of extended and repeated dosing on these pharmacokinetic and inflammatory outcomes after TBI.

**Significance Statement:**

To our knowledge, this is the first pharmacokinetic analysis of morphine and its metabolite morphine-3-glucuronide (M3G) following traumatic brain injury (TBI). This research provides the first evidence that morphine and M3G show increased systemic and brain concentrations following experimental TBI, with an acute rise in M3G’s brain/plasma ratio. Although no exacerbation of acute TBI-induced inflammation was observed with either morphine or M3G, the impact of longer, more frequent dosing needs evaluation because its longer administration could exacerbate TBI’s neuroinflammatory response.

## 1 Introduction

In 2019 alone, the United States recorded 280,000 hospitalizations and over 60,000 fatalities linked to severe traumatic brain injury (TBI).^1^ Among those that survive their injury, a considerable number of patients will endure temporary or long-term disabilities.^2^, ^3^, ^4^

The clinical management of severe TBI focuses on stabilizing the patient and mitigating secondary injury in the intensive care unit.^5^ Morphine is commonly administered in the intensive care unit intravenously to stabilize mechanically ventilated patients.^6^ Morphine is primarily hepatically metabolized to morphine-3-glucuronide (M3G) and morphine-6-glucuronide (M6G).^7^ Despite M6G being a minor metabolite (5%–10%), it has been a major focus of research interest because it is a more potent analgesic than the parent compound. M3G is the major metabolite (50%–70%) but lacks analgesic effects.^8^

Recent reports show an increase in interleukin (IL)-1B, IL-6, and tumor necrosis factor (TNF)-*α* after morphine and/or M3G administration in rodents and clinically in the contexts of acute inflammatory brain injury.^9^, ^10^, ^11^, ^12^, ^13^, ^14^ Under normal circumstances, morphine crosses the BBB, but M3G is impermeable owing to its more hydrophilic nature.^15^ However, when the BBB is compromised post-TBI, M3G may gain access to brain parenchyma, where it has been shown to interact with neuroinflammatory receptors located on astrocytes and microglia.^16^ Astrocytes and microglia produce proinflammatory cytokines, which subsequently can activate additional glial cells, thus propagating the inflammatory response. Neuroinflammation, occurring as a secondary injury response after TBI, can worsen patient outcomes when exacerbated. The microglial protein ionized calcium–binding adaptor molecule (IBA)-1 and astrocytic glial fibrillary acidic protein (GFAP) have been associated with an increased response of proinflammatory mediators.^17^^,^^18^

The pharmacokinetics of morphine and M3G after TBI have not been thoroughly investigated to date. While some clinical studies have investigated the influence of TBI pathophysiology on morphine clearance,^19^, ^20^, ^21^ their findings have been inconsistent possibly because of underlying variations in patients, type of injuries, and underlying comorbidities.

Understanding morphine disposition in the context of TBI requires knowledge of the key enzymes and transporters involved in its ADME (absorption, distribution, metabolism, and excretion) processes. In rats, morphine is primarily metabolized by UGT2B1 to M3G, which accounts for 80%–90% of metabolism, while M6G formation is negligible (<1%). Roughly 20% of morphine is converted to normorphine via CYP3A. In humans, metabolism is primarily through UGT2B7, producing both M3G and M6G, with M6G contributing significantly to analgesia (∼10%–15%). Transport across the BBB involves P-glycoprotein (efflux), organic cation transporters and OATP2B1 (uptake), while MRP2/3 are responsible for exporting glucuronide metabolites. Renal elimination is the primary route of excretion for both morphine and its metabolites. These processes may be altered in the setting of TBI, potentially influencing central drug and metabolite exposure. Determining whether these changes exist is a necessary first step before investigating the mechanisms responsible for altered disposition.

The primary objective of this study is to characterize the effect of TBI on systemic and central nervous system (CNS) exposure of M3G following morphine intravenous bolus administration using the well-characterized rodent controlled cortical impact (CCI) model. This model was chosen because it replicates key features of moderate-to-severe focal contusion injury seen in clinical TBI, including BBB disruption and neuroinflammation, allowing us to evaluate morphine and its metabolites under these conditions. The secondary objective is to determine whether markers of neuroinflammation and systemic inflammation are altered following morphine intravenous bolus administration in the setting of TBI. In addition, M3G was directly administered to determine the clearance of M3G and examine the effect of M3G alone. Results will elucidate pharmacokinetic changes in clearance and M3G exposure after TBI, highlighting how these could potentially worsen neuroinflammation acutely.

## 2 Materials and Methods

### 2.1 Animals

Experiments used 8–10-week-old, 290–340-g, male Sprague-Dawley rats (Invigo). All experimental protocols were completed in compliance with ARRIVE guidelines and received approval from the University of Pittsburgh Institutional Animal Care and Use Committee, in compliance with the guidelines outlined in the National Institutes of Health’s Guide for the Care and Use of Laboratory Animals. The animal housing facilities accommodated a maximum of 2 rats per cage, with lighting set on 12:12 light/dark photoperiod, and animals had continuous access to food and water. Veterinary technicians and laboratory staff conducted daily health assessments and husbandry.

### 2.2 Jugular catheter surgery

Anesthesia was induced with 4% isoflurane and 2:1 N_2_O:O_2_ mixture using a ventilated anesthesia chamber. Following endotracheal intubation, anesthesia was maintained using 2% isoflurane in 2:1 N_2_O:O_2_ mixture via mechanical ventilator. Rats were placed within a stereotaxic frame, with their core body temperature maintained at 37 °C using a homeothermic heating pad and rectal thermal probe. Following aseptic preparation of the site, a surgical incision was performed to isolate the right jugular vein, and a 3-F polyurethane catheter (Instech) was carefully inserted into the vein. Sutures were used to secure the catheter to surrounding muscle tissue. The remaining portion of the catheter was externalized through a subcutaneous tunnel at the back of the neck to ensure secure placement. To ensure the catheter lines were free of any obstructions, they were flushed with 300 *μ*L of saline before being capped with bone wax.

### 2.3 Experimental design

Using the CCI injury protocol,^22^ this study aimed to assess differences in the pharmacokinetics of an intravenous bolus of morphine and M3G, along with the acute neuroinflammatory response after TBI. All experimental procedures on rats were conducted simultaneously in 1 anesthetic setting. Rats were randomized to receive either injury or sham control surgery and treatment with morphine, M3G, or saline. Pharmacokinetic experiments followed a randomized 1 × 2 design, with experimenters assessing concentrations blinded to the specific conditions of injury and agent administered.

For pharmacodynamic analysis, samples obtained during the pharmacokinetic experiments were repurposed to establish correlations between drug area under the plasma concentration–time curve (AUC_0–last_) values and inflammation markers (GFAP, IBA1, IL-6, KC-GRO, and TNF-*α*). A 2 × 2 design was adopted to evaluate the combined effects of injury and treatment, involving morphine versus saline and M3G versus saline. These experiments were conducted in a randomized fashion with the experimenters blinded to the specific conditions of injury and treatment.

### 2.4 CCI injury

Rats were randomized to receive either sham control or CCI surgery. A midline incision was performed, and a 7-mm craniotomy was created over the right parietal cortex between bregma and lambda centered 5-mm lateral from the sagittal suture and the dura mater was exposed. CCI was conducted using a specialized apparatus (Pittsburgh Precision Instruments), equipped with a small-bore (1.975 cm) double-acting pneumatically driven cylinder with a 5.0-cm stroke. An impactor tip, with a diameter of 6 mm was positioned to generate a tissue deformation of 2.5 mm at a velocity of 4 m/s, with a dwell time of 100 milliseconds. Sham control surgery animals underwent anesthesia and surgical procedures identical to those of the CCI group but were not subjected to injury. After either sham control surgery or CCI injury, the scalp incision was sutured, anesthesia was discontinued, and the righting time was recorded and monitored. Upon regaining ambulatory status, the animals were returned to their respective home cages.

### 2.5 Administration of morphine intravenous bolus and serial plasma collection

A morphine intravenous bolus of 2.5 mg/kg was selected for administration. Previous studies revealed that larger doses of morphine administered to animals lead to extended periods of respiratory depression and, in certain instances, hindered functional recovery.^23^^,^^24^ Morphine sulfate pentahydrate (Sigma) was dissolved in 0.9% saline and subsequently filtered through a sterile 0.22-*μ*m membrane.

To investigate pharmacokinetics, the rats were categorized into 2 groups (sham control surgery vs CCI injury). All animals were randomized to receive either CCI injury or sham control surgery, and either a 2.5-mg/kg i.v. bolus of morphine or volume equivalent saline via jugular catheter (*n* = 6/group). The volume of intravenous boluses were 1.0 ± 0.15 mL and delivered at a steady rate for over 60 seconds. Blood samples (300 *μ*L) were collected via the jugular catheter at specific time intervals including (0, 4, 10, 30, and 60 minutes and 2, 4, 12, and 20 hours postinjury). The 0-minute sample was collected immediately after intravenous bolus morphine administration, which occurred following the CCI or sham procedure. The selection of these time points was made to facilitate a comprehensive systemic characterization of both *α* and terminal elimination phases of morphine.^25^ After drug administration and blood collection, an equivalent volume of saline (300 *μ*L) was used to rinse the jugular catheter to replace volume withdrawn and to minimize the potential for clot formation. The jugular catheter was then capped with bone wax after every time point.

The blood samples were transferred to EDTA-coated tubes and centrifuged (12,000*g* for 20 minutes) to isolate plasma. These plasma samples were then stored at −80 °C until drug concentration and inflammation markers analysis. To explore the pharmacodynamic response, the experiment was replicated with an equivalent volume of saline and given in 2 separate groups (*n* = 6) receiving either sham control surgery or CCI injury.

### 2.6 Administration of M3G intravenous bolus and serial plasma collection

A 2.5-mg/kg dose was selected to achieve a similar AUC_o–∞_ that was observed in a clinical study in which a M3G intravenous bolus was given to healthy volunteers^26^ (Supplemental Figure A). M3G (Lipomed) was dissolved in 0.9% saline and filtered through a sterile 0.22-*μ*m membrane. The 2.5-mg/kg M3G intravenous bolus was delivered in the same manner as morphine to rats in groups (*n* = 6) to receive either sham or CCI injury. The volume of intravenous boluses were 1.0 ± 0.15 mL and delivered at a steady rate for over 60 seconds. Blood samples (300 *μ*L) were collected via the jugular catheter at specific time intervals including (0, 4, 10, 30, and 60 minutes and 2, 4, 20, and 24 hours postinjury and drug administration). The 0-minute sample was collected immediately after intravenous bolus M3G administration, which occurred following the CCI or sham procedure. Blood samples at the terminal time point were also collected and separated into plasma in the same manner, before being stored for analysis.

### 2.7 Administration of morphine intravenous bolus for morphine and M3G measurement in brain 1 hour after CCI

The experimental design for assessing morphine and M3G concentrations in the brain was duplicated from the previous design measuring plasma, with the sole alteration being the terminal time point, which was adjusted to 1 hour postinjury. Two groups, each comprising 6 rats (*n* = 6), were randomly assigned to either the CCI injury or sham control surgery conditions. Subsequently, all rats received a 2.5-mg/kg i.v. bolus of morphine. Blood samples were obtained via the jugular catheter at the 1-hour mark, immediately before the administration of the euthanizing agent. Following this, tissue from the ipsilateral cortex was dissected and promptly frozen through flash freezing for subsequent liquid chromatography-mass spectrometry (LC-MS) analysis. *T*_max_ for M3G was calculated as the median of observed values across animals in each group.

### 2.8 Sample preparation for LC/MS-MS measurement

#### 2.8.1 Plasma

The sample preparation for plasma followed a validated protocol established in our laboratory.^27^ Protein precipitation was achieved by combining 100 *μ*L of plasma with acetonitrile and the internal standard. The resulting mixture centrifuged at room temperature (12,000*g*, 8 minutes). The supernatant was transferred to a new vial and subsequently dried at 38 °C under a nitrogen flow. The dried samples were reconstituted with 75 *μ*L of mobile phase (90% [0.15% formic acid in water] with 10% [acetonitrile]) and vortex mixed. After reconstitution, samples were centrifuged under the conditions mentioned previously, and 7.5 *μ*L was injected onto the LC/MS-MS system for analysis.

#### 2.8.2 Brain tissue homogenate

Dissected ipsilateral (same side as injury or sham control surgery) cortical brain samples were weighed before homogenization in 1 mL of water. A 100-*μ*L aliquot of homogenate was processed using the same procedure as for plasma. A validation was conducted to assess the accuracy and precision of the assay in brain tissue matrix (Supplemental Section C).

### 2.9 LC-MS/MS instrumentation and analytical conditions

The LC/MS-MS method described previously was used for this study.^27^ To achieve chromatographic separation of analytes, an Acquity BEH C18 column (2.1 × 100 mm, 1.7 *μ*m) equipped with a BEH C18 VanGuard precolumn (2.1 × 5 mm, 1.7 *μ*m) was used on an ultrahigh-performance liquid chromatography system, Thermo Vanquish (Thermo Scientific). The method entailed a gradient elution with a duration of 10 minutes and a sample volume of 7.5 *μ*L. The mobile phases used were 0.15% formic acid in water (mobile phase A) and acetonitrile (mobile phase B). The initial conditions of the gradient consisted of 95% A for the first minute, followed by a gradual decrease to 25% A at 3.7 minutes. These gradient conditions were maintained until 4.5 minutes to ensure elution of all analytes. Subsequently, the column was rinsed with 60% A until 7.9 minutes, after which it was equilibrated back to the initial conditions until the 10-minute mark. The flow rate for the entire injection was maintained at 0.2 mL/min, with the column temperature set at 50 °C and the autosampler temperature at 10 °C.

For the detection and quantification of analytes, a TSQ Altis triple quadrupole mass spectrometer (Thermo Scientific) equipped with a heated electrospray ionization source was used. Analytes were detected in positive ionization mode with the following parameters: a spray voltage of 4000 V, a vaporization temperature of 275 °C, sheath gas pressure (in arbitrary unit [au]) set at 35, and auxiliary gas pressure (arbitrary units) at 7. Analytes were monitored using the selected reaction monitoring mode, with the following parameters configured: chromatographic peak width of 6 seconds, scan time of 0.05 seconds, and Q1 and Q3 (full width at half maximum) set at 0.70. This LC-MS assay was validated for measurement of morphine (0.5–125 ng/mL), M3G (1–500 ng/mL), and M6G (1–500 ng/mL) in plasma and brain.

### 2.10 Pharmacokinetic analysis

A noncompartmental pharmacokinetic analysis was performed using Phoenix WinNonlin 8.4 (Certara) to determine the pharmacokinetic parameters of morphine and its metabolite, M3G. The concentration of analyte at time zero (*C*_0_) was calculated by extrapolating the natural log-transformed values of the first 2 plasma analyte concentrations back to time zero following intravenous administration. The elimination rate constant (*β*) was determined by plotting log-transformed concentration versus time and calculating the slope of the last 3 points in the terminal linear phase. Clearance (CL) was calculated using noncompartmental methods based on the equation CL = dose/AUC_0–∞_. The terminal half-life (*T*_1/2_) was derived using the equation *T*_1/2_ = ln 2/*β*. Volume of distribution at steady state (*V*_ss_) was calculated using the following equation: *V*_ss_ = (dose × AUMC_0–∞_)/(AUC_0–∞_).^2^ AUMC_0–*t*_ was determined by multiplying each time point with its corresponding concentration. These values were then plotted against time, and linear trapezoidal integration was used to calculate AUMC_0–*t*_. Where to calculate AUMC_*t*–∞_, both AUMC_0–*t*_ and AUMC_*t*–∞_ needs to be summed together. The AUC_0–*t*_ from zero to *T*_last_ was determined by linear trapezoidal integration and AUMC_*t*–∞_ was calculated using the following equation: AUMC_*t*–∞_ = (*C*_last_ × *T*_last_)/*β* + (*C*_last_/*β*^2^). Formation clearance of M3G was estimated using a method adapted from Derendorf and Schmidt,^28^ using data from intravenous bolus administrations of morphine and M3G and the equation: formation clearance of M3G = [(M3G from morphine AUC_0–20_)/(morphine AUC_0–20_)] × (M3G clearance). Pharmacokinetic analysis was repeated after applying exclusion criteria, ensuring consistency of parameters using a sparse sampling approach and excluding rats with an adjusted *R*^2^ ≤ 0.8 or fewer than 3 terminal phase points.

### 2.11 Plasma, cerebrospinal fluid and tissue collection for biomarker analysis

To facilitate correlations between biomarker levels and morphine or M3G exposure, the same rats that were used for the pharmacokinetic analysis were also used in the biomarker analysis. Therefore, a 24-hour time point was selected for analyzing neuroinflammatory and inflammatory markers, aligning with our secondary objective to complement the primary goal of determining pharmacokinetics. Twenty-four hours following the injury or sham surgery, animals were euthanized by an overdose of 100 mg/kg, sodium pentobarbital intraperitoneally (Fatal-plus; Vortech pharmaceuticals). Cerebrospinal fluid (CSF) was obtained via cisterna magna. Transcardial collection of blood samples were collected in EDTA-coated tubes, as previously detailed, and processed for plasma. Subsequently, animals underwent transcardial perfusion with saline, followed by 10% neutral buffered formalin (Fisher Scientific). Brains were then postfixed for 24 hours in 10% neutral buffered formalin, dehydrated by immersion in a solution of 30% sucrose in 0.1 M phosphate-buffered saline for 48 hours at 4 °C and embedded in Tissue-Tek O.C.T compound (Sakura Finetek) for sectioning into 35-*μ*m-thick coronal sections using a cryostat (Leica Microsystems).

### 2.12 Immunohistochemistry analysis of IgG, GFAP, and IBA1

For immunohistochemical staining, hippocampal tissue sections covering the region between −3.2 and −4.0 mm bregma, which encompassed the site of injury, were selected for analysis. Immunohistochemical staining was conducted on free-floating tissue sections. For each protein of interest, a single section per animal, was rinsed with a 0.1 M Tris-buffered saline (TBS) buffer and blocked with a solution of 10% normal goat serum and 0.1% Triton X-100 in 0.1 M TBS for a duration of 1 hour. Sections were then incubated overnight at 4 °C with the primary antibodies diluted in 0.1% Triton X-100 in 0.1 M TBS (rabbit anti-GFAP: 1:5000; Millipore; RRID:AB_2109645; rabbit anti-IBA1: 1:2000; Wako; RRID:AB_839504), horseradish peroxidase secondary antibodies were applied in accordance with the kit instructions (1:500; Vector; RRID:AB_2336820, RRID:AB_2336821), washed, visualized with diaminobenzidine (Vector; RRID:AB_2336382), mounted onto Superfrost Plus slides (Fisher Scientific), cover-slipped using Permount medium (Fisher Scientific). For each protein, all tissues from each group (sham control surgery vs CCI injury) and treatment (saline, morphine, and M3G) were processed together. This allowed for controlled diaminobenzidine reaction time and maintained consistent exposure for all tissues within each protein analysis. For IgG immunohistochemistry, the same procedure was followed in which a biotinylated secondary antibody recognizing Rat IgG (1:500; Vector; RRID:AB_2336823) antibody was used to recognize extravasated IgG. The imaging process was facilitated using a C2 Nikon 90i microscope. Individual imaging scans of stained brain slices at 10× magnification were stitched into a comprehensive image, encompassing the cortex, hippocampus, and thalamus. Microscope settings were held consistent for all images for a protein of interest. Using ImageJ, images were converted to 16-bit grayscale and inverted to determine mean pixel intensity of the ipsilateral hippocampus, as boundaries defined by rat brain atlas.^29^

### 2.13 Assessment of inflammatory markers

Inflammatory markers (interferon gamma, IL-1*β*, IL-4, IL-5, IL-6, KC-GRO, IL-10, IL-13, and TNF-*α*) were measured in plasma and CSF at the 24-hour time point in duplicate using the Multi-Spot rat proinflammatory panel 2 kit (Meso Scale Discovery) using manufacturer’s instructions. Values were reported if they fell within the calibration curve on each plate and exhibited a coefficient of variation within each duplicate of <25%. For each marker, if both duplicates met the reporting criteria, they were averaged to provide the representative value for that specific marker analysis in the animal. In the event only 1 replicate met this value, the rat was excluded from the analysis of that marker. Outlier tests were not performed because the variability in inflammatory markers can reflect heterogeneous responses post-CCI, thus all data points within the measurable range of the multiplex standard curves are included.

### 2.14 Data analysis and statistical methods

To ensure sufficient statistical power in our study, a priori group size of 6 rats was determined for each group to complete all analyses, including pharmacokinetic and pharmacodynamic analysis. The data analysis was carried out using GraphPad Prism 10 software. Welch *T* test was used for comparison of CCI vs sham in pharmacokinetic analysis. For determining pharmacodynamic results, group comparisons were performed using a 2-way ANOVA. For pharmacodynamic analysis, all values that were detected in both replicates, and meet coefficient of variation criteria mentioned previously, a minimum of *n* = 5 was needed to perform statistical significance testing. For correlation analysis, a nonparametric Spearman correlation coefficient test was used. The results are expressed as mean values ± SD, and statistical significance was established at a threshold of *P* < .05.

## 3 Results

### 3.1 Intravenous bolus pharmacokinetics of morphine and M3G

**Fig. 1:**
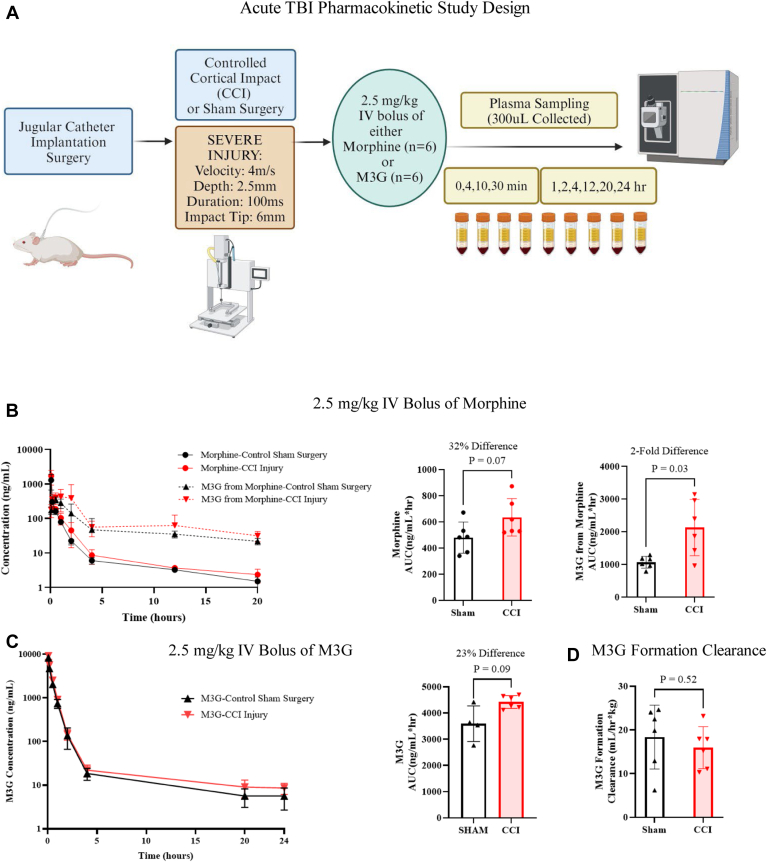
Pharmacokinetic analysis of morphine and M3G following CCI over 24 hours. (A) Schematic of study design. (B) Mean concentration of morphine and M3G in plasma (±SD) of CCI and sham rats (*n* = 6) with bar graphs displaying AUC_0–20_ of morphine and M3G from a 2.5-mg/kg morphine intravenous bolus. (C) Mean concentration of M3G in plasma (±SD) of CCI and sham rats (*n* = 6) with bar graphs displaying AUC_0–24_ of M3G from a 2.5-mg/kg M3G intravenous bolus. (D) Bar graph displaying M3G formation clearance after a 2.5-mg/kg morphine intravenous bolus in acute CCI and sham rats (*n* = 6) after 24 hours. For (B) and (D), Welch *T* test was used for comparison of CCI vs sham in pharmacokinetic analysis to determine significance.

We investigated differences in morphine and M3G pharmacokinetics between animals receiving CCI injury and control sham injury (Fig. 1, A). Calculated pharmacokinetic parameters for both morphine and M3G intravenous boluses are summarized in Table 1. Following the administration of a 2.5-mg/kg morphine intravenous bolus, no significant differences in morphine clearance were observed in animals that were CCI-injured compared with those who received sham control surgery (Fig. 1, B; see Supplemental Section E for inset of 0–4 hours). The AUC_0–24_ of M3G after intravenous bolus administration of morphine was increased 2-fold (*P* = .03) in injured animals compared with sham surgery (Fig. 1, B).

**Table 1:** Noncompartmental parameter estimates for 2.5-mg/kg morphine and 2.5-mg/kg M3G intravenous bolus administration 24 h after CCI in male Sprague-Dawley rats

	Sham (Mean ±SD) *n* = 6	CCI (Mean ± SD) *n* = 6	*P*
**Pharmacokinetic parameters from 2.5-mg/kg morphine intravenous bolus**			

C_0_ (ng/mL)	3747.0 ± 3179.4	5398.0 ± 9178.2	.52
AUC_0–20_ (ng/mL × h)	479.7 ± 146.7	614.4 ± 164.4	.07
AUC_0–∞_ (ng/mL × h)	480.0 ± 119.8	635.3 ± 143.5	.16
CL (mL/min × kg)	89.0 ± 25.7	67.9 ± 13.7	.13
T_1/2_ (h)	8.1 ± 2.2	7.3 ± 1.2	.47
V_ss_ (L/kg)	15.8 ± 4.0	11.7± 2.2	.40

**Pharmacokinetic parameters from 2.5mg/kg M3G intravenous bolus**			

C_0_ (ng/mL)	8978.0 ± 1162.8	12,601.0 ± 1482.2	.02
AUC_0–24_ (ng/mL × h)	3592.0 ± 828.7	4421.0 ± 245.4	.09
AUC_0–∞_ (ng/mL × h)	3653.0 ± 836.5	4551.0 ± 195.7	.07
CL (mL/min × kg)	11.7 ± 2.7	9.2 ± 0.5	.11
T_1/2_ (h)	9.9 ± 1.7	11.8 ± 3.9	.31
Vss (L/kg)	1.2 ± 1.2	1.3 ± 0.3	.62

**Pharmacokinetic parameters of M3G from 2.5-mg/kg morphine intravenous bolus**			

*C*_max_ (ng/mL)	372.9 ± 61.0	674.4 ± 409.6	.13
T_max_ (h)	0.4 ± 0.2	1.1 ± 0.7	.07
AUC_0–20_ (ng/mL × h)	1038.0 ± 141.6	1936.0 ± 701.0	.03
Formation CL (mL/min × kg) based on 0–20 h	16.0 ± 6.0	18.4 ± 7.3	.62

To investigate the M3G clearance specifically, a separate 2.5-mg/kg i.v. bolus of M3G was administered to a distinct cohort of CCI injury and sham control surgery animals. No significant changes in M3G clearance were observed (Fig. 1, C).

Using the pharmacokinetic results from both the morphine and M3G intravenous bolus experiments, formation clearance was calculated. Formation clearance of M3G was not significantly altered following injury (Fig. 1, D). Overall, AUC_0–∞_ for every rat was <10% of AUC_0–∞_, demonstrating adequate sampling and exposure characterization. A sparse resampling modeling approach analysis further confirmed these results (Supplemental Section B).

### 3.2 Brain concentrations of morphine and M3G and their systemic contributions

**Fig. 2:**
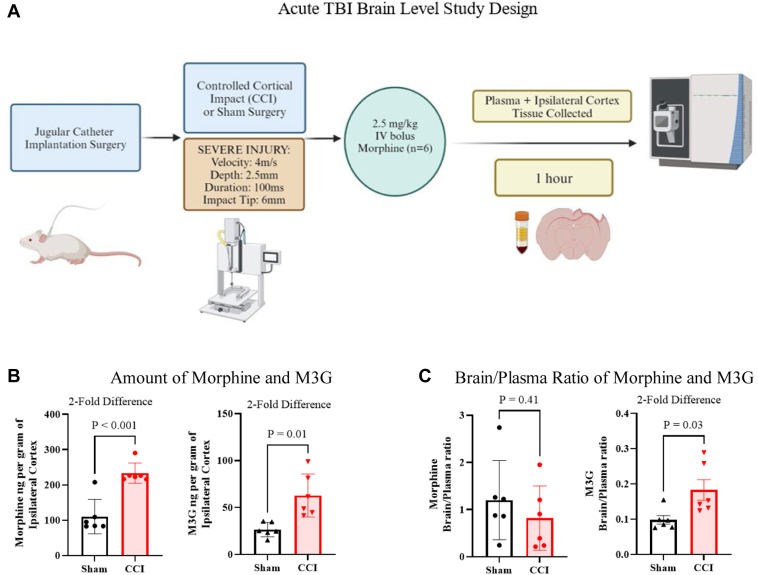
Brain concentrations and brain/plasma ratio of morphine and M3G following 2.5-mg/kg morphine intravenous bolus administration at 1 hour after CCI. (A) Schematic of study design. (B) Morphine and M3G mean amounts in the ipsilateral cortex (side of injury or sham surgery; ±SD) 1 hour after CCI and sham surgery rats (*n* = 6) from a 2.5-mg/kg morphine intravenous bolus. (C) Bar graphs of morphine and M3G mean brain/plasma ratio (±SD) 1 hour after CCI and sham surgery rats (*n* = 6) from a 2.5-mg/kg morphine intravenous bolus. For (B) and (C), Welch *T* test was used for comparison of CCI vs sham in pharmacokinetic analysis to determine significance.

To assess whether CCI resulted in an acute increased brain exposure to morphine and M3G, concentrations were measured 1 hour post-CCI (Fig. 2, A). This time was selected based on the maximum concentration (*C*_max_) of M3G observed following morphine administration in the previous experiment (Table 1). A 2-fold increase (morphine: *P* = .001; M3G: *P* = .01) in the amount of both analytes per gram of tissue was evident in CCI-injured rats than that in sham control surgery (Fig. 2, B). The morphine brain/plasma ratio did not change (Fig. 2, C). However, M3G brain/plasma ratio demonstrated a 2-fold increase (*P* = .03) in CCI-injured animals. Detailed results from this experiment are summarized in Table 2.

**Table 2:** Morphine and M3G amounts and brain/plasma estimates from a 2.5-mg/kg intravenous bolus administration 1 h after CCI in male Sprague-Dawley rats

2.5-mg/kg Morphine Intravenous Bolus at 1 h in Ipsilateral Cortex	Sham (Mean ±SD) *n* = 6	CCI (Mean ±SD) *n* = 6	*P*
Amount of morphine (ng/g)	110.5 ± 48.7	233.4 ± 28.4	<.01
Amount of M3G (ng/g)	26.5 ± 7.6	62.9 ± 23.0	.01
Brain blood ratio morphine	1.20 ± 0.84	0.82 ± 0.68	.41
Brain blood ratio M3G	0.10 ± 0.03	0.18 ± 0.07	.03

### 3.3 Evaluation of pharmacodynamic responses following morphine and M3G intravenous bolus administration

#### 3.3.1 Immunohistochemistry

**Fig. 3:**
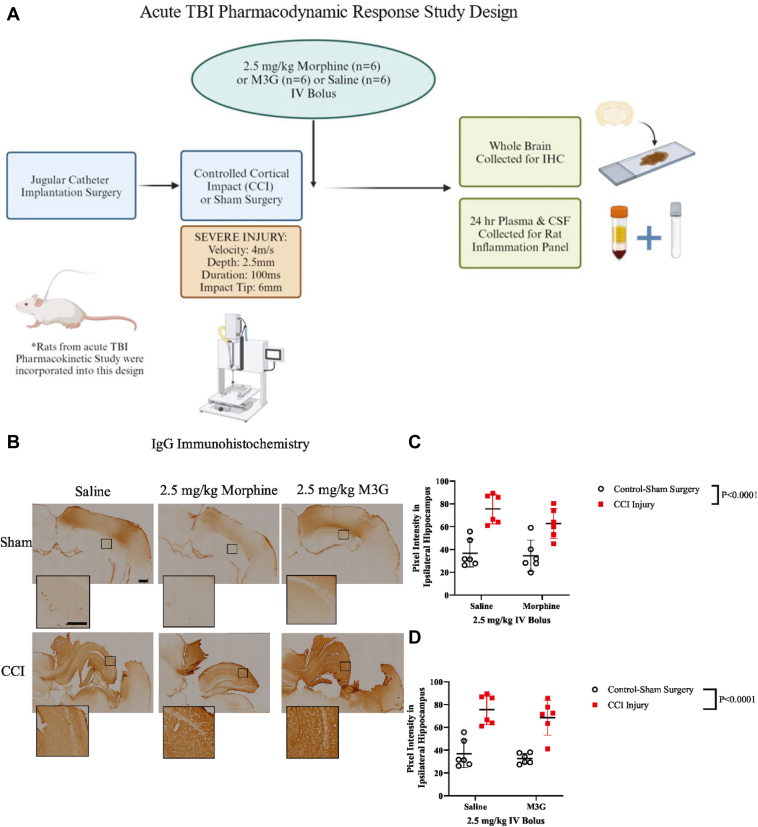
Hippocampal IgG extravasation at 24 hours after CCI. (A) Schematic of study design to complete immunohistochemistry in brain and fluid tissues completed in animals from the pharmacokinetic analysis. (B) Representative immunohistochemical images of IgG in the ipsilateral hippocampus (side of injury or sham surgery). Scale bar of lower magnitude images equal to 1 mm while higher magnitude images are equal to 200 *μ*m. (C) Plots of 2-way ANOVA comparison of saline vs 2.5-mg/kg morphine in measurement of IgG immunoreactivity with (±SD). (D) Plots of 2-way ANOVA comparison of saline vs 2.5-mg/kg M3G in measurement of IgG immunoreactivity with (±SD).

To determine whether neuroinflammation response is heightened owing to increased drug exposure, inflammatory biomarkers in brain, CSF, and plasma were assessed in multiple tissue outcomes (Fig. 3, A).

IgG extravasation was used as a marker for detecting BBB breakdown as an intact BBB restricts its movement into the parenchyma. We used pixel intensity measurements of IgG staining within the ipsilateral hippocampus to determine whether M3G-affected or morphine-affected BBB permeability jointly with the effects from injury. Two-way ANOVA results revealed a significant injury effect—saline vs morphine (Fig. 3, C); CCI-injured: 69.25 ± 6.44 au vs sham control surgery: 35.65 ± 1.16 au; saline vs M3G (Fig. 3, D); CCI-injured: 72.18 ± 3.52 au vs sham control surgery: 34.77 ± 2.04 au—in all rats, irrespective of treatment. No treatment effect or interaction was observed between saline and morphine or saline and M3G. These data show that the BBB breakdown was consistent in all CCI-injured rats with no difference observed for saline, morphine, or M3G treatment status. There was no significant interaction effect observed between injury and treatment for IgG extravasation analysis.

**Fig. 4:**
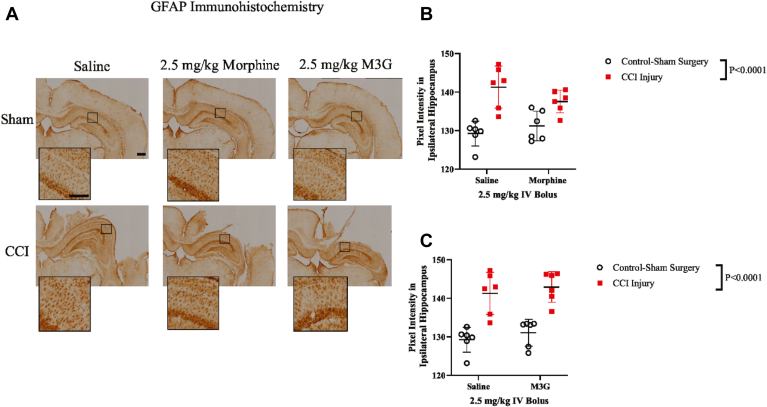
Hippocampal GFAP immunoreactivity at 24 hours after CCI. (A) Representative immunohistochemical images of GFAP in the ipsilateral hippocampus (side of injury or sham surgery). Scale bar of lower magnitude images equal to 1 mm, while higher magnitude images are equal to 200 *μ*m. (B) Plots of 2-way ANOVA comparison of saline vs 2.5-mg/kg morphine in measurement of GFAP IgG immunoreactivity with (±SD). (C) Plots of 2-way ANOVA comparison of saline vs 2.5-mg/kg M3G in measurement of GFAP IgG immunoreactivity with (±SD).

Subsequently, we examined GFAP, a well-established biomarker indicative of astrocytic reactivity. We measured pixel intensity of GFAP staining within the ipsilateral hippocampus (Fig. 4, A). Statistical analysis revealed a significant effect of injury, with increased intensity observed in all rats, regardless of treatment (Fig. 4, B: CCI-injured, 139.4 ± 2.66 au vs sham control surgery, 130.2 ± 1.39 au; Fig. 4, C: CCI-injured, 141.3 ± 1.15 au vs sham control surgery, 129.2 ± 1.28 au). However, no overall treatment effect was observed.

**Fig. 5:**
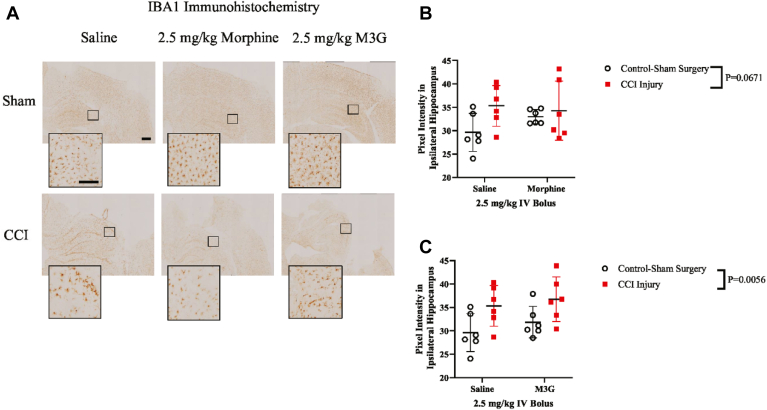
Hippocampal IBA1 immunoreactivity at 24 hours after CCI. (A) Representative immunohistochemical images of IBA1 IgG in the ipsilateral hippocampus (side of injury or sham surgery). Scale bar of lower magnitude images equal to 1 mm, while higher magnitude images are equal to 200 *μ*m. (B) Plots of 2-way ANOVA comparison of saline vs 2.5-mg/kg morphine in measurement of IBA1 IgG immunoreactivity with (±SD). (C) Plots of 2-way ANOVA comparison of saline vs 2.5-mg/kg M3G in measurement of IBA1 IgG immunoreactivity with (±SD).

Consequently, our analysis progressed to the examination of IBA1, a marker for microglial activity. Pixel intensity measurements of IBA1 staining in the ipsilateral hippocampus were examined and quantified (Fig. 5, A and B). There was a significant injury effect when saline was compared against M3G (CCI-injured: 36.03 ± 0.71 au vs sham control surgery: 30.74 ± 1.11 au) (Fig. 5, C). No treatment effect or interaction was observed between saline and morphine or saline and M3G.

#### 3.3.2 Neuroinflammation measurement in 24-hour CSF

**Fig. 6:**
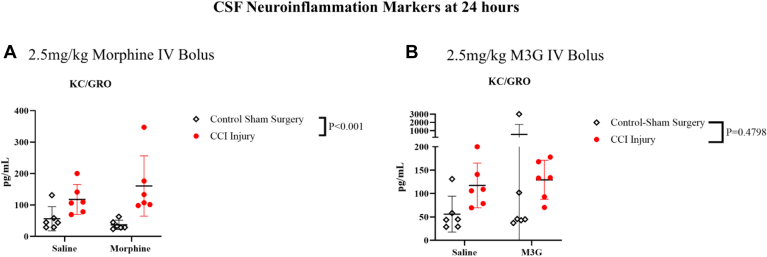
Plots of 2-way ANOVA comparison with (±SD) of saline vs 2.5-mg/kg morphine (A) and saline vs 2.5-mg/kg M3G (B) in measurement in of KC-GRO using a Mesoplex Rat Inflammatory Panel.

Next, we measured a panel of inflammatory markers in CSF to evaluate the effect of morphine and M3G on markers of neuroinflammation after CCI. Our exclusion criteria were applied, and only KC-GRO met the criteria to complete statistical analysis. However, results from all inflammatory markers in the panel are included in Supplemental Section D. A significant injury effect with increased levels of KC-GRO was observed when comparing morphine against saline (Fig. 6, A) (CCI-Injured: 138.90 ± 21.50 pg/mL vs Sham control surgery: 46.42 ± 9.58 pg/mL). However, no significant injury effect was observed when comparing M3G with saline (Fig. 6, B). Interactions with injury for morphine vs saline and M3G vs saline were not significant.

#### 3.3.3 Inflammatory measurement in 24-hour plasma

**Fig. 7:**
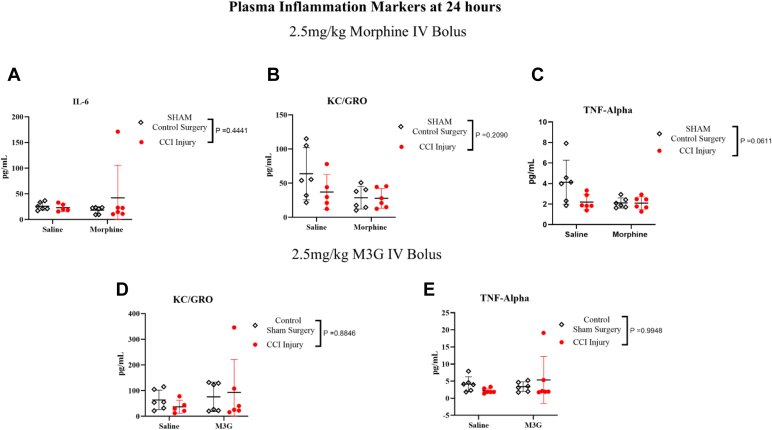
Plots of 2-way ANOVA comparison with (±SD) of saline vs 2.5-mg/kg morphine (A–C) and saline vs 2.5-mg/kg M3G (D,E) in measurement of IL-6 (A), KC-GRO (B,D), TNF*α* (C,E), using a Mesoplex Rat Inflammatory Panel.

Gaining insight into the inflammatory response is crucial for elucidating its impact on neuroinflammation. Following the application of exclusion criteria, statistical analysis was exclusively feasible for IL-6 (only morphine vs saline) (Fig. 7, A), KC-GRO (Fig. 7, B and D), and TNF*α* (Fig. 7, C and E). Findings for the other markers can be found in Supplemental Section D. No significant interactions, injury effects, or treatment effects were observed, except a modest injury effect showed increased KC-GRO in rats treated with morphine compared with those with saline.

#### 3.3.4 Correlation analysis between M3G AUC_0–24_ in CCI rats and neuroinflammation

**Fig. 8:**
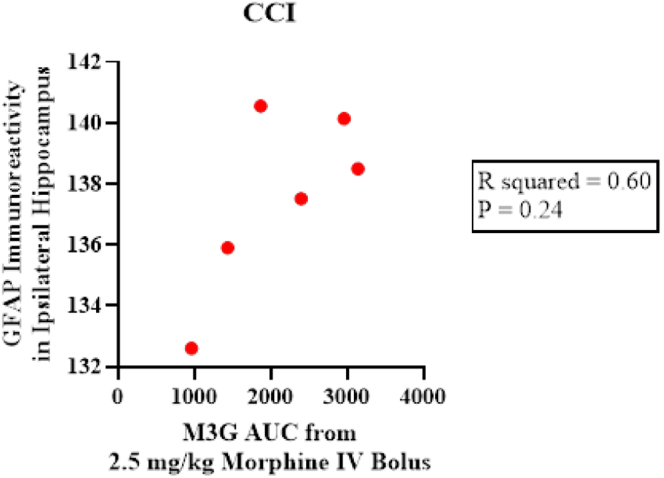
Correlation plot of injured rats of M3G AUC_0–24_ from 2.5-mg/kg intravenous bolus of morphine (*n* = 6) compared with GFAP pixel intensity of the ipsilateral hippocampus of the same injured rat. Nonparametric Spearman correlation coefficient test was used to determine statistical significance.

The data from our GFAP analysis suggested a trend in the interaction with injury and morphine. To evaluate whether M3G exposure from morphine is related to this, further analysis was performed. We completed correlation plots to explore the within-subject relationship between GFAP as an indicator of astrocytic reactivity and M3G exposure from formation from morphine (Fig. 8). Notably, the pixel intensity of GFAP staining in the ipsilateral hippocampus of injured rats treated with a 2.5-mg/kg i.v. bolus of morphine exhibited a modest, but nonsignificant correlation with M3G AUC_0-last_.

## 4 Discussion

The primary objective of this study was to characterize the effect of TBI on systemic and CNS exposure of M3G following morphine intravenous bolus administration using the well-characterized rodent CCI model. Evaluating the PK of morphine and its metabolite M3G is important because understanding how TBI influences their systemic and CNS exposure could have significant implications for patient outcomes. Alterations in the pharmacokinetics of these compounds following TBI may impact their effectiveness and safety, influencing clinical decision-making and treatment strategies. This approach enables a clearer understanding of how TBI affects morphine and M3G pharmacokinetics, which is crucial for optimizing treatment in patients with TBI.

Disruption of the BBB is a key secondary injury feature of CCI,^30^ which recapitulates vascular disruptions reported in patients afflicted with a severe contusion TBI. Our key findings show CCI-injured rats exhibited increased systemic concentrations of morphine and M3G. Additionally, CCI-injured rats had increased concentrations of both morphine and M3G in the brain at a singular time point of 1 hour, with only M3G showing an increased brain to plasma concentration ratio. This time point was selected based on the systemic *C*_max_.

Studies characterizing the PK of morphine and M3G following intravenous bolus and intraperitoneal administration show similar parameter estimates, once dosing, bioavailability, and experimental variables are considered.^31^^,^^32^ Notably, our study implemented a comprehensive and robust sampling strategy distributed over a 24-hour period that enabled estimation of the AUC_0–20_ and clearance. In contrast, previous literature commonly evaluated shorter sampling intervals of 0.5–4 hours, which could compromise the characterization of clearance and the *β* (terminal phase) of drug elimination.

The increase in the concentrations of M3G following CCI is unexpected because there is no significant change in morphine clearance. One possible explanation is altered shunting between metabolic pathways. In rats, approximately 80%–90% of morphine is converted to M3G via UGT2B1, while M6G formation is negligible (<1%), which aligns with our undetectable M6G levels. Additionally, normorphine accounts for ∼20% of morphine metabolism via CYP3A. We did not measure normorphine because our LC-MS assay was optimized for clinical use where normorphine comprises only ∼4% of metabolism.^31^^,^^33^ Previous studies investigating changes in CYP enzymes shortly after TBI have reported decreased expression 24 hours after CCI in rats,^34^ although the specific impact of TBI on the CYP3A variant has not been examined in this time point. Considering our current observations and the application of this clinical assay to animal studies, these findings support future incorporation of normorphine into the assay.

It is noteworthy that kidney function has not been characterized temporally in TBI. Clinical studies have highlighted occurrences of concurrent hypotension and severe TBI.^35^ Neijmann et al^36^ investigated kidney function in rats subjected to mild and moderate brain trauma, measuring creatinine concentrations and acute kidney injury biomarkers over 24 hours. Although no significant differences in creatinine concentrations were observed, distinct variations were noted in neutrophil gelatinase-associated lipocalin and kidney injury molecule-1 concentrations among the groups.^36^ However, the kidney function in the context of this CCI rat model remains largely uncharacterized. It is possible that renal clearance might be disrupted during CCI, potentially contributing to the accumulation of M3G. To determine whether this change stemmed from M3G formation clearance, we used a surrogate method based on parameters derived from the intravenous bolus of morphine and M3G. However, the preferred method for determining M3G formation clearance involves measuring M3G in urine. Unfortunately, urine collection was not completed for this study. Future studies should include urine analysis and will also need to examine kidney function in the rat CCI model.

Brain transporters may explain the observed 2-fold increase in morphine exposure within the brain. Previous studies have shown increased P-glycoprotein efflux activity after TBI or morphine administration.^37^^,^^38^ However, other transporters have not been evaluated extensively in the context of TBI, which may counter the effects of P-glycoprotein efflux. Another potential explanation is central metabolism. The brain/plasma ratio of M3G alongside a nonsignificant decrease in morphine suggests that morphine may be metabolized to M3G within the CNS. UGT enzymes, although primarily hepatic, have been identified in rodent and human brain tissue and shown to glucuronidate morphine in vitro.^39^^,^^40^ Supporting this, in vitro studies and our previous work have demonstrated morphine-to-M3G conversion in brain-resident cells.^41^ However, no studies to date have quantified centrally formed M3G following morphine administration post-CCI.

To evaluate BBB permeability in our study, IgG extravasation was completed. The substantial injury effect observed in our study was consistent with findings in the literature, particularly at the 24-hour time point.^42^ IgG normally has poor BBB penetration. By quantifying pixel intensity within the ipsilateral hippocampus, we were able to assess BBB permeability, a parameter influenced by the extent of injury.^43^

Given the previous associations of M3G with astrocyte signaling,^44^ we conducted immunohistochemical analysis of GFAP. The results indicated a significant injury effect, although treatment with either morphine or M3G did not exacerbate this response. In the study by Chen et al.,^45^ GFAP activity in the ipsilateral hippocampus of rats has shown a significant injury effect 24 hours after CCI and increase progressively, peaking at 72 hours. Examining the 72-hour time point after injury could provide additional insight into whether morphine or M3G could exacerbate astrocytic reactivity postinjury into the days postinjury.

We also assessed IBA1 owing to the involvement of microglia in neuroinflammatory response, which have been shown to have an injury effect with increased activity at 24 hours.^46^ Immunohistochemistry analysis reveals a clear injury effect comparing saline with M3G and a potential trend compared with morphine. The observed trend in morphine-treated rats may be due to morphine’s known influence on microglia expression, enhancing it at 24 hours in normal rats.^47^ Further analysis is necessary for a comprehensive understanding of its impact.

To further explore neuroinflammation and systemic inflammation, we examined downstream markers linked to neuroinflammation namely, IL-1*β* and TNF*α*.^48^ Additionally, we assessed markers linked to M3G exposure and had been previously studied in vitro, revealing elevated concentrations of IL-1*β* and IL-10.^16^^,^^49^ Unfortunately, statistical testing could not be conducted on these analytes because they did not meet the inclusion criteria. It is plausible that their concentrations at 24 hours might not be sufficiently elevated, suggesting that examining later time points, could clarify the neuroinflammation time course seen in other studies.^50^

The results from our neuroinflammation panel indicated a significant injury effect, in the morphine administration study. The increased levels of this marker were consistent with previous literature, demonstrating increased KC-GRO at 24 hours post-CCI.^51^ Collectively, these findings suggest that the downstream markers neuroinflammation may not be heightened at this time point in CSF or plasma. Additionally, TBI-induced alterations in opioid receptor expression or responsiveness (eg, MOR downregulation) may influence pharmacodynamic outcomes and warrant further investigation.^52^, ^53^, ^54^

In our experiment, M3G formed from morphine was not correlated to GFAP immunoreactivity. Although previous proteomic studies in cultured human astrocytes exposed to M3G showed increased signaling,^44^ our analysis was conducted in an in vivo TBI model. The lack of GFAP exacerbation may be partly due to our use of a single intravenous bolus. At the current dosing, M3G concentrations exceed the proposed *K*_m_ (∼600 ng/mL)^55^ for only ∼10 minutes, which may be insufficient to drive a measurable astrocytic response. Whether neuroinflammation requires prolonged exposure or a higher *C*_max_ remains unclear. Clinically, patients often receive repeated boluses or continuous infusions of morphine. Given our findings, opioids such as fentanyl, which do not generate M3G, may be more appropriate in the setting of BBB disruption. Alternatively, coadministering neuroinflammatory inhibitors with morphine could mitigate M3G’s proinflammatory effects while preserving analgesia.

## 5 Conclusion

In conclusion, our study revealed differences in injury related to morphine and M3G exposure both systemically and in the brain after morphine administration. This study focused on administration of a single intravenous bolus dose and revealed that it did not result in exacerbated secondary responses after TBI. Considering previous work on neuroinflammatory responses by M3G, it is plausible that multiple intravenous bolus doses or infusion of morphine, which is clinically relevant, may lead to a heightened accumulation or response. These findings support the need for future research to explore the effects of multiple boluses and infusion dosing at later time points postinjury.

## Conflict of interest

S.C. and T.N. report financial support was provided by NIH. J.B. and S.C. report financial support was provided by The Pittsburgh Foundation. All other authors declare no conflicts of interest.
