# Supplementary material for: Pharmacokinetic analysis of morphine-3-glucuronide after acute morphine intravenous bolus administration to rats with traumatic brain injury
Source: J Pharmacol Exp Ther. 2025 Jun 25;392(8):103645. doi: 10.1016/j.jpet.2025.103645 (PMC12489372; doi:10.1016/j.jpet.2025.103645)
Supplement: Supplementary Material [file mmc1.docx]

**Article Title:** Pharmacokinetic Analysis of Morphine-3-Glucuronide After Acute Morphine IV Bolus Administration to Rats with Traumatic Brain Injury

**Authors:** Jonathan Birabaharan, Jeremy Henchir, Sarah Svirsky, Thomas D. Nolin, Philip E. Empey and Shaun W. Carlson

**Journal Title:** Journal of Pharmacology and Experimental Therapeutics

**Manuscript Number:** JPET-D-25-00116R1

Supplemental Section A


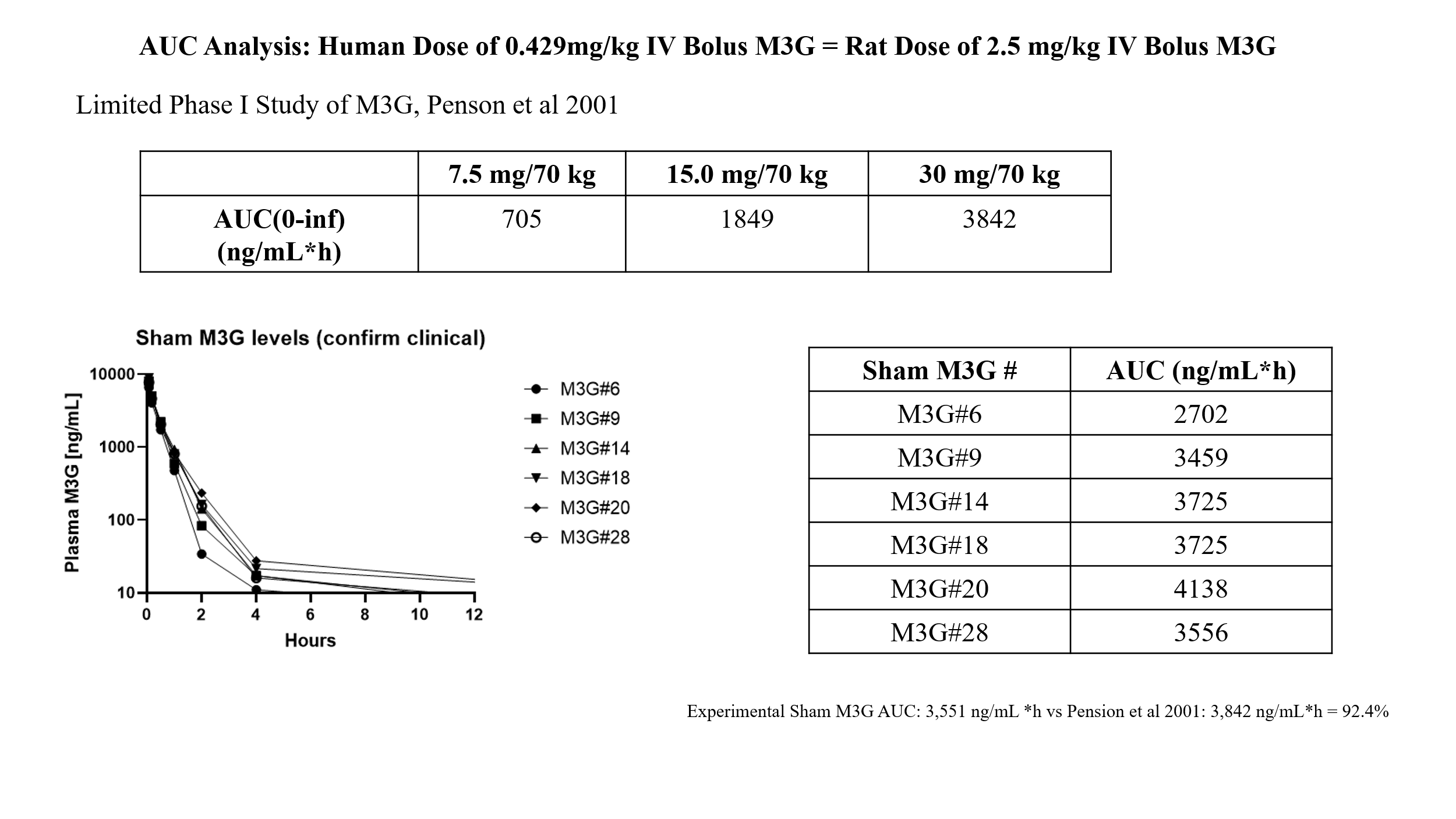


In investigating the impact of M3G administered independently on the neuroinflammatory response in TBI, we aimed to replicate a clinical scenario with a single IV bolus. Drawing from Penson et al. 2001, who explored three M3G dosages in healthy volunteers, we successfully mirrored their clinical dose (30mg/70kg). The clinical data showing AUC values with 0.4290mg/kg M3G dose were utilized from Penson et al. 2001 for modeling goals. Comparison of the AUC with the sham control surgery rats receiving 2.5mg/kg in our study exhibited similar AUC values to those observed in the clinical study, enhancing the translatability of our findings.

Supplemental Section B

Sparse sampling approach of noncompartmental parameter estimates for 2.5 mg/kg morphine and 2.5 mg/kg M3G IV bolus administration 24 hours after CCI in male Sprague-Dawley Rats.

| **Pharmacokinetic parameters from 2.5mg/kg morphine IV Bolus** | **Sham**  **N=1** | **CCI**  **N=1** |
| --- | --- | --- |
| C_0_ (ng/mL) | 3399.1 | 5324.3 |
| AUC_0-20_ (ng/mL*hr) | 470.2 | 631.1 |
| AUC_0-∞_ (ng/mL*hr) | 487.7 | 660.2 |
| CL (mL/min*kg) | 85.4 | 63.1 |
| T_1/2_ (hr) | 8.1 | 8.6 |
| Vss (L/kg) | 13.8 | 11.3 |
|  |  |  |
| **Pharmacokinetic parameters from 2.5mg/kg M3G IV Bolus** | **Sham**  **N=1** | **CCI**  **N=1** |
| C_0_ (ng/mL) | 11986.0 | 131.0 |
| AUC_0-24_ (ng/mL*hr) | 3672.0 | 4346.0 |
| AUC_0-∞_ (ng/mL*hr) | 3760.0 | 4483.3 |
| CL (mL/min*kg) | 11.1 | 9.3 |
| T_1/2_ (hr) | 10.9 | 12.9 |
| Vss (L/kg) | 1.3 | 1.3 |
|  |  |  |
| **Pharmacokinetic Parameters of M3G from 2.5mg/kg Morphine IV Bolus** | **Sham**  **N=1** | **CCI**  **N=1** |
| C_max_ (ng/mL) | 176.4 | 423.1 |
| T_max_ (hr) | 0.5 | 1.0 |
| AUC_0-20_ (ng/mL*hr) | 1039.0 | 2036.0 |
| Formation CL (mL/min*kg) based on 0-20 hours | 24.5 | 30.0 |

The table above displays results from sparse sampling via Phoenix WinNonlin 8.4 (Certara, Princeton, New Jersey). All rats (n=6 per group) were included, preventing statistical testing. Results confirmed with the original analysis, which excluded rats not meeting inclusion criteria.

Supplemental Section C


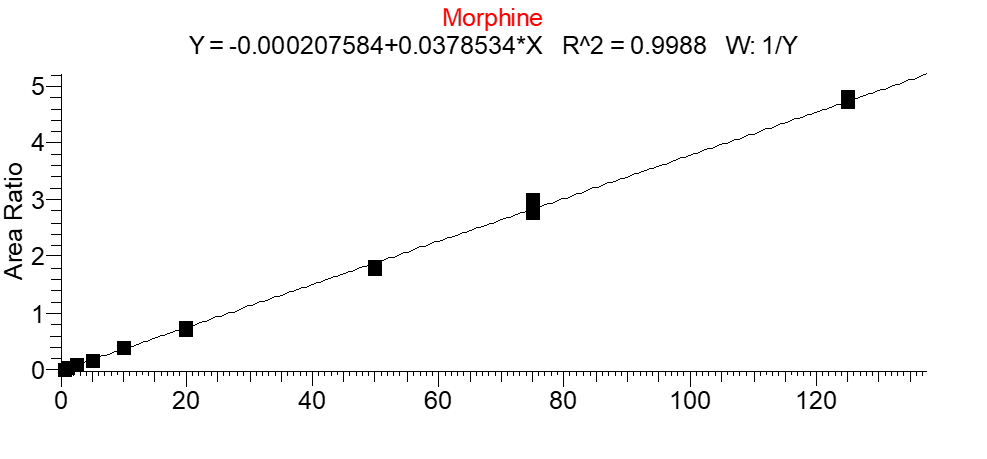


| Morphine | Brain Replicate 1 | | Brain Replicate 2 | | Brain Replicate 3 | | Brain Replicate 4 | | Brain Replicate 5 | |
| --- | --- | --- | --- | --- | --- | --- | --- | --- | --- | --- |
|  | Conc | %CV | Conc | %CV | Conc | %CV | Conc | %CV | Conc | %CV |
| HQC(100ng/mL) | 109.1 | 9% | 104.1 | 4% | 104.0 | 4% | 104.0 | 4% | 102.7 | 3% |
| MQC(30ng/mL) | 28.5 | -5% | 28.2 | -6% | 28.0 | -7% | 27.4 | -9% | 27.0 | -10% |
| LQC(1.5ng/mL) | 1.7 | 13% | 1.5 | 0% | 1.6 | 7% | 1.4 | 4% | 1.6 | 4% |


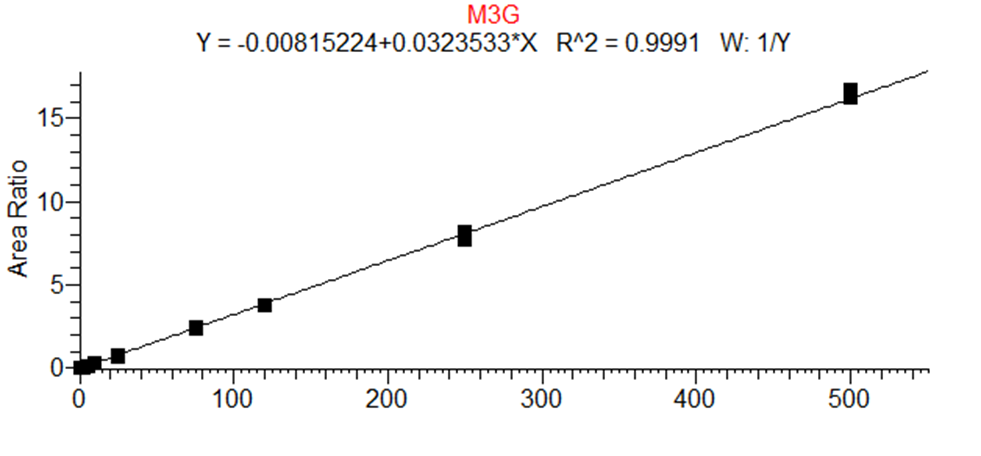


| M3G | Brain Replicate 1 | | Brain Replicate 2 | | Brain Replicate 3 | | Brain Replicate 4 | | Brain Replicate 5 | |
| --- | --- | --- | --- | --- | --- | --- | --- | --- | --- | --- |
|  | Conc | %CV | Conc | %CV | Conc | %CV | Conc | %CV | Conc | %CV |
| HQC(400ng/mL) | 438.0 | 9% | 422.8 | 6% | 432.9 | 8% | 421.9 | 5% | 417.6 | 4% |
| MQC(60ng/mL) | 65.5 | 9% | 60.3 | 1% | 59.2 | -1% | 59.9 | 0% | 61.3 | 2% |
| LQC(3ng/mL) | 3.0 | 0% | 2.9 | -1% | 3.0 | 1% | 3.1 | 2% | 2.9 | -1% |

To quantify morphine and M3G in rat brain tissue, we enhanced our LC/MS-MS assay sensitivity. Recognizing the need for improved M3G sensitivity, we refined the method with two extra centrifuge steps, modifying our approach from previously described, Birabaharan et al. 2022. This adjustment increased M3G sensitivity to 1-500 ng/mL, addressing concerns from our initial analysis near the lowest limit of quantification (LLOQ). A mini-validation ensued with three quality control (QC) levels, five replicates each, in rat brain tissue spiked with varying analyte concentrations. A duplicate nine-point curve, using rat brain tissue from a different source than the QC preparation, demonstrated accurate and precise measurement of both morphine (0.5-125 ng/mL) and M3G (1-500 ng/mL) in rat brain tissue.

Supplemental Section D


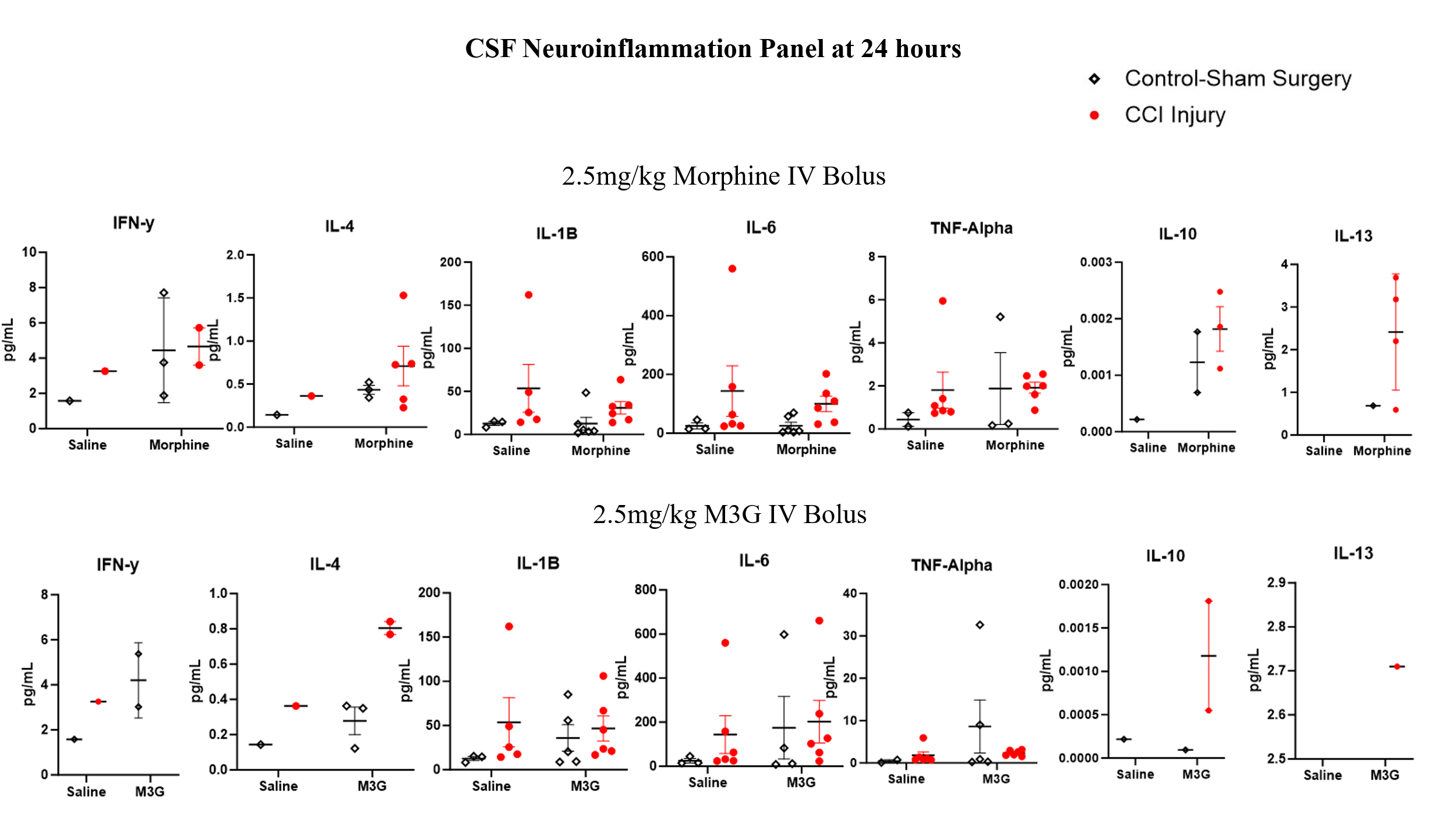


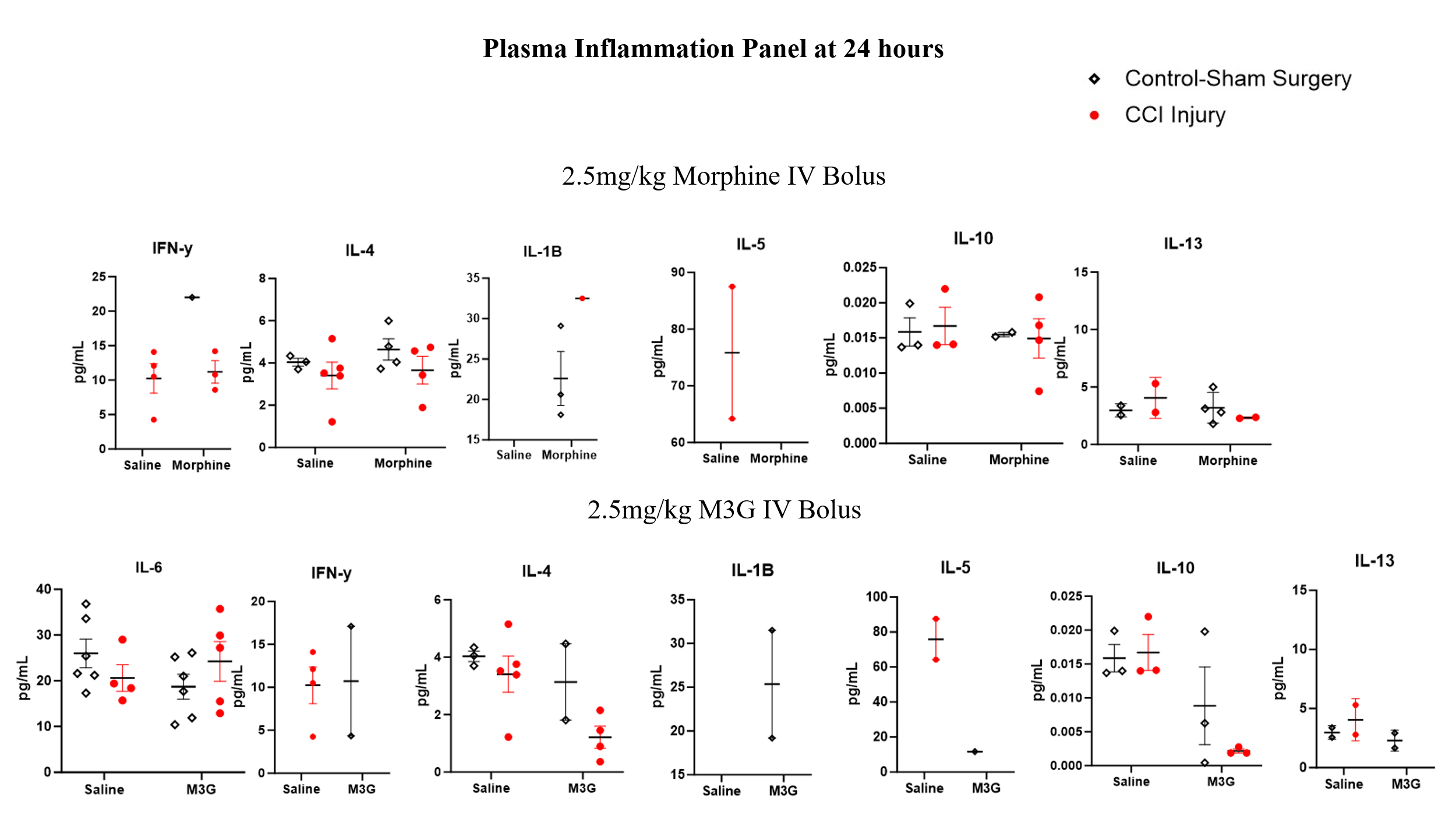


Utilizing a Mesoplex rat inflammatory panel for nine markers, we analyzed duplicate plasma and CSF samples. Statistical testing was exclusively applied to markers detected in both replicates, with a CV<25%, and a group size of at least n=5. The criteria applied were as follows: values were reported if they fell within the calibration curve on each plate and exhibited a coefficient of variation (CV) within each duplicate of less than 25%. If both duplicates for a marker met these criteria, their average was taken as the representative value for that marker analysis in the animal. If only one replicate met the criteria, the rat was excluded from the analysis for that marker.

Supplemental Section E


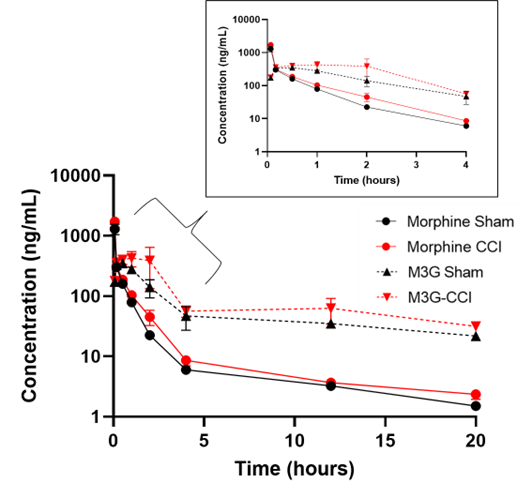


Zoomed-in pharmacokinetic profiles of morphine and M3G over the first 4 hours following IV bolus administration. Concentration-time curves are shown for morphine (2.5 mg/kg; circles) and M3G (2.5 mg/kg; triangles) in both sham (black) and CCI-injured (red) rats. This inset highlights the early distribution and elimination phase of each compound, corresponding to the critical time window following injury and dosing. Data are presented as mean ± SEM (n = 6 per group).
